# A Sustainable Strategy for Gastrointestinal Nematode Control in Sheep

**DOI:** 10.3390/vetsci13010104

**Published:** 2026-01-21

**Authors:** Lorella Giuliotti, Maria Novella Benvenuti, Angelo Gazzano, Valentina Gazzano, Giorgia Romeo, Fabio Macchioni

**Affiliations:** 1 Department of Veterinary Sciences, University of Pisa, 56124 Pisa, Italy; lorella.giuliotti@unipi.it (L.G.); novella.benvenuti@unipi.it (M.N.B.); angelo.gazzano@unipi.it (A.G.); fabio.macchioni@unipi.it (F.M.); 2 Office for Hunting and Fishing Activities of Grosseto Regional Administration, Via C. Colombo 5, 58100 Grosseto, Italy; giorgina.romeo@regione.toscana.it

**Keywords:** ovine, resilience, parasite resistance, wool cortisol, EPG, farm sustainability

## Abstract

Gastrointestinal nematodes (GIN) represent a major constraint to sheep production, and sustainable alternatives to routine anthelmintic use are increasingly required. This study compared two parasite control strategies in Zerasca sheep: routine albendazole treatment administered twice yearly (T) and a long-term non-chemical approach based on rotational grazing (relocation to a new pasture when grass height fell below 10 cm) combined with quarterly veterinary monitoring (NT). Twenty-four adult ewes (*n* = 12 per group) were monitored over an eight-month period. Mean faecal egg counts (EPG) differed significantly between groups (T: 210 ± 78; NT: 529 ± 89; *p* = 0.0007). In group T, EPG values decreased markedly 7 days after treatment but increased again by 150 days, resulting in no persistent differences between groups over time. Despite higher parasite burdens, NT sheep showed significantly higher body condition scores compared with treated animals (3.00 ± 0.61 vs. 2.51 ± 0.53; *p* = 0.0014). Haematological parameters were largely comparable between groups, although mild reductions in RBC, HGB, and HCT were observed in both. Treated sheep exhibited higher AST activity (*p* < 0.0001) and transient increases in ALT and BUN following treatment. Hair cortisol concentrations did not differ significantly between groups. Overall, these findings suggest that a non-chemical parasite management strategy, when combined with controlled grazing and veterinary monitoring, may maintain acceptable parasite levels while supporting body condition and stable welfare indicators, potentially reducing reliance on routine anthelmintic treatments.

## Simple Summary

Sustainable parasite control in sheep must reconcile environmental protection with animal health and welfare. This study compared two gastrointestinal nematode management strategies in an endangered local sheep breed: routine anthelmintic treatments versus a long-term non-chemical approach based on rotational grazing and veterinary monitoring. Although treated animals showed a temporary reduction in parasite egg counts shortly after deworming, this effect was short-lived and disappeared within five months. Sheep managed without routine anthelmintics maintained moderate parasite burdens without clinical disease and displayed significantly better body condition scores throughout the study. Haematological parameters were largely comparable between groups, while treated animals showed higher liver enzyme activity and transient metabolic alterations following drug administration. Long-term stress indicators, assessed by hair cortisol, did not differ between strategies, suggesting similar welfare levels. Overall, the results indicate that non-chemical parasite management, when combined with careful monitoring, can maintain animal health and welfare while reducing drug use and potential environmental contamination. This approach represents a viable and sustainable alternative to routine anthelmintic treatments in extensive sheep farming systems.

## 1. Introduction

Gastrointestinal nematodes (GIN) are recognized as some of the most prevalent and significant pathogens affecting the gastrointestinal tract in domestic ruminants globally [1]. In infected animals, these parasites can lead to severe clinical manifestations as well as notable declines in production in sub clinically infected subjects, resulting in significant economic losses [2]. GIN infections presently cause significant financial losses to the global small ruminant livestock sector, amounting to millions of dollars each year [3].

Decreased productivity can result from a combination of anorexia and diminished efficiency in resource utilization for production objectives [4]. GIN infections can result in significant alterations within the digestive tract of small ruminants, including heightened cell turnover, modifications in permeability, variations in pH, changes in secretory activities, and reduced gastric acid production [5]. As a result, GIN infections can lead to an imbalance of nutrients and adversely affect feed intake, growth and weight gain, fertility, as well as the quality and quantity of milk [6,7].

Historically, parasite control in livestock has predominantly relied on the use of antiparasitic drugs. While various anthelmintics possess distinct mechanisms of action, all function to either induce parasite death or impair motility through drug-induced paralysis [8]. The benzimidazole family represents the most extensive chemical group of anthelmintic drugs, featuring widely utilized derivatives such as fenbendazole, albendazole, and oxfendazole. The cytoskeleton is compromised by these drugs through a selective interaction with β-tubulin, impacting cellular structure and mitosis [8].

The extensive use of veterinary drugs in livestock therapy significantly impacts the environment and poses considerable risks to ecosystems [9]. These substances are introduced into the environment through excretions in pastures or via manure from treated animals. The application of dung, advocated for sustainable agriculture due to its role in mineral or nitrogen sequestration, poses the risk of disseminating veterinary pharmaceuticals onto agricultural land. Over 50% of unmetabolized drugs ultimately enter soil or water [10] and may be taken up by plants that can metabolize these compounds and accumulate them in their tissues [11,12]. Moreover, certain veterinary drugs can adversely impact plant physiology and endogenous metabolism [13,14,15]. Lastly, the aboveground parts of plants that contain accumulated drugs and their metabolites may be ingested by invertebrates, farm animals, and humans, thereby presenting significant risks to ecosystems [13].

Anthelmintics are specifically designed to be toxic to helminths, and their harmful effects on invertebrates have been well documented [16,17,18,19]. Regarding wild and farm animals, anthelmintics at low environmental concentrations do not exhibit acute toxicity; however, prolonged exposure, such as through the consumption of contaminated plants, may pose chronic toxicity risks. Moreover, the ingestion of contaminated plants by animals infected with helminths could promote the development of drug resistance due to interactions between residual anthelmintics and parasites [20].

The swift emergence of anthelmintic resistance is acknowledged as a worldwide threat to livestock production [21,22]. Resistance is defined as the heritable capacity of a parasite to withstand a typically effective dosage of anthelmintic agents. A parasite is deemed resistant if it endures exposure to the standard recommended dose of an anthelmintic, with this survival trait being inherited by its offspring [23]. Parasite resistance results from genetic mutations that enable parasites to withstand anthelmintic (dewormer) drugs, which are increasingly ineffective due to their frequent and indiscriminate application [24]. GIN parasite resistance is a complex issue that encompasses the parasite’s capacity to endure pharmacological treatments [25] as well as the host’s inherent genetic resilience to infection. This phenomenon raises sustainability concerns by undermining the long-term effectiveness of available drugs and increasing the environmental burden due to chemical residues and treatment frequency [26].

Sustainable nematode control in sheep requires an integrated approach that includes grazing management strategies such as rotation, rest periods, avoidance of overgrazing, and multi-species grazing. Additionally, genetic selection through breeding for parasite resistance [27], and alternative control methods, including bioactive plants with condensed tannins, copper oxide wire particles, and nematophagous fungi, can be components of this strategy [28,29,30,31,32,33,34].

Regarding grazing management, potential interventions include pasture rotation, which involves frequently moving sheep to new pastures to disrupt the parasite’s life cycle, and rest, which allows for sufficient recovery periods for pastures to eliminate larvae. In temperate climates, a rest period of 100 days or more can be effective [35]. Additionally, rotational multi-species grazing involves grazing a different species, such as cattle, on a pasture after it has been grazed by sheep [36]. Other animals will ingest the sheep-specific parasite larvae, and their own parasites will not develop in the sheep. It is important to avoid overgrazing [37], as most infective larvae are concentrated in the bottom four inches of grass. Moreover, ensure clean pastures for vulnerable animals by grazing young or low-immunity animals on areas that have not been recently grazed by contaminated adult animals [38].

Integrated management practices and selective treatment approaches are increasingly advocated as alternatives to diminish dependence on chemical interventions [39]. It is essential to emphasize that worms cannot be eliminated from the host or the environment. Infections can be mitigated through an understanding of parasite dynamics and prevalence. This knowledge is crucial for the implementation of integrated control strategies and for minimizing reliance on chemical treatments [40]. The concept of resilience is increasingly pertinent to sustainable livestock systems. Resilience denotes an animal’s capacity to sustain productivity and overall health in the presence of parasitic infections [41]. Resistance pertains to the reduction in parasite burden, whereas resilience emphasizes the animal’s ability to manage infection without a substantial decline in performance [42]. The promotion of resilient animals and systems enhances production sustainability by reducing drug dependency, minimizing costs, and bolstering the natural adaptive capacity of flocks. Research on host resistance is being conducted in animals such as sheep, utilizing breeding programs to select individuals and breeds with enhanced natural abilities to combat parasites, identifiable through traits such as low fecal egg counts [43].

Ultimately, the research seeks to provide insights into how sustainable and resilient parasite control practices can be implemented in modern sheep farming to enhance both environmental and economic sustainability [27].

This study fits within this line of research and aims to compare two farming systems in Zerasca sheep adopting contrasting parasite management strategies: one based on regular anthelmintic treatments and the other relying on a non-chemical approach.

## 2. Materials and Methods

The study was carried out in accordance with European Commission regulations and approved by the Ethics Committee of the University of Pisa (Italy), decision no. 31/2022. It took place between August 2022 and April 2023 and involved Zerasca sheep, a native breed classified as endangered. The research was conducted on two farms located in the Zeri district (Massa Carrara, Italy) at an altitude of 900 m above sea level (44°19′ N, 9°47′ E). Both farms were situated within the same valley and experienced comparable climatic conditions. No new animals had been introduced into the flocks during the 12 months preceding the study. Each flock comprised approximately 60 individuals and shared similar management practices, on approximately 14 ha of natural pasture (stocking density ≈ 4 ewes/ha). The primary source of nourishment for the sheep consisted of natural pasture, including polyphyte meadow grasses, shrubs, and bushes. In addition, animals were supplemented daily with polyphyte meadow hay and corn, according to pasture availability and physiological stage.

Both farms provided salt licks to ensure adequate mineral intake.

The first farm implemented a conventional gastrointestinal parasite control regimen, administering albendazole (Sverminator^®^-FATRO S.p.A., Ozzano dell’Emilia, Bologna, Italy) *per os* at a dose of 3.75 mg/kg of body weight twice annually, in autumn and spring, to control GIN. In contrast, the second farm had not administered any gastrointestinal anthelmintic treatments over the past ten years, adhering instead to a non-chemical parasite management approach. This strategy relies on rotational grazing and regular monitoring of GIN (every three months) under the supervision of a veterinarian.

Grazing rotation was implemented by moving the animals to a new grazing area when, following browsing, grass height was reduced to approximately 10 cm in height [44]. In fact, it has been demonstrated that infective third-stage larvae (L3) of trichostrongylid nematodes are unevenly distributed along the vertical profile of the herbage, with the highest concentrations occurring near the base of the sward [45].

In cases of increased egg per gram (EPG), the veterinarian assessed the need for targeted anthelmintic treatment of individual animals showing a reduction in Body Condition Score (BCS).

For this study, twenty-four adult ewes (*n* = 12 per group) were monitored over an eight-month period. The ewes were randomly selected from each flock to form two experimental groups: group T composed of animals treated with anthelmintic drug; and group NT composed of animals that did not usually receive a vermifuge, ensuring homogeneity in age (3–6 years) and parity (≥2) to minimize potential bias. The investigation was conducted from August 2022 to April 2023. A veterinarian assessed the general health status of all animals during each sampling session. The presence of ectoparasites is regularly monitored in the flock, with treatment of particularly infested animals, but no antiparasitic treatments were carried out during the research.

**Figure 1:**
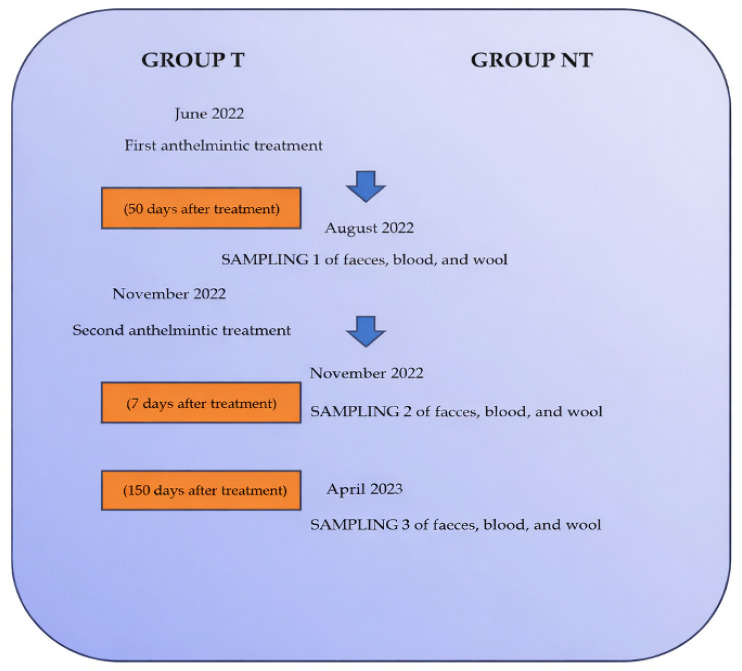
Timeline of the two anthelmintic administrations in the treated group (T) and of the three blood, faecal, and wool sampling time points in both treated (T) and non-treated (NT) groups during the study period.

Over the study period, two anthelmintic treatments were administered to the Group T flock in June and November. Three sets of blood, faecal, and wool samples were collected simultaneously from both treated (T) and non-treated (NT) groups, according to the study timeline reported in Figure 1. Sampling was performed under different climatic conditions: June (temperature 22–28 °C; mean relative humidity, RH, 60%), November (7–12 °C; RH 80%), and April (12–18 °C; RH 70%). This sampling strategy allowed evaluation of the effects of anthelmintic treatment in the short, medium, and long term, as well as under varying climatic conditions.

Faecal samples were collected directly from the rectal ampulla and analyzed using the McMaster technique [46] to estimate the faecal egg count of gastrointestinal strongyles, expressed as EPG. Samples were processed within 24 h of collection and subsequently stored at 4 °C until analysis. EPG values were categorized into three levels following Ambrosi’s classification [47]: level 1 = 0–300 EPG, level 2 = 301–600 EPG, and level 3 = >600 EPG.

Pooled fecal samples were subjected to coproculture according to Henriksen and Korsholm [48,49], and third-stage larvae were identified morphologically. Moreover, the samples were examined by macroscopic inspection for the presence of cestode proglottids [50].

Blood samples were collected from the jugular vein using Vacutainer^®^ tubes (Becton, Dickinson & Co., Milan, Italy) and stored in a refrigerated container (+5 °C) until delivery to the laboratory. Complete blood counts were performed at the Veterinary Clinical Pathology Unit, Department of Veterinary Sciences (University of Pisa, Italy), using a CELL-DYN 3500^®^ automated haematology analyser (Abbott, Minneapolis, MN, USA). The following haematological parameters were assessed: red blood cell count (RBC), haematocrit (HCT), haemoglobin (HGB), mean corpuscular volume (MCV), mean corpuscular haemoglobin (MCH), mean corpuscular haemoglobin concentration (MCHC), reticulocytes (RETIC), white blood cells (WBC), neutrophils (NEU), lymphocytes (LYM), monocytes (MON), eosinophils (EOS), basophils (BAS), and platelets (PLT).

Serum was separated, frozen, and sent to the Istituto Zooprofilattico Sperimentale delle Regioni Lazio e Toscana (IZSLT) for the analysis of selected metabolic and immunological parameters. These included: alanine aminotransferase (ALT), aspartate aminotransferase (AST), blood urea nitrogen (BUN), β-hydroxybutyric acid (BHBA), non-esterified fatty acids (NEFA), total proteins (TP), creatinine (Creat), calcium (Ca), chloride (Cl), phosphorus (P) and potassium (K), analysed using an automatic biochemical analyser (Olympus AU 400, San Diego, CA, USA).

Oxygen free radicals (OFR) were quantified using a commercial colorimetric method (Diacron International S.r.l., 58100, Grosseto, Italy).

Wool samples were collected from the shoulder area, between the scapular bones, and as close as possible to the skin, using scissors. The wool samples were stored in aluminum foil and labelled envelopes at room temperature until analysis at the ETOVET Laboratory, Department of Veterinary Sciences, University of Pisa (Italy). Wool samples were processed using the ELISA method described by Mariti et al. [51] to evaluate hair cortisol.

Serum cortisol concentrations were measured according to the method previously described by Gazzano and colleagues [52].

BCS was evaluated by one of the authors (M.N.B.) who has extensive experience in the field, according to the scale of Russel et al. [53], ranging from one (emaciated) to five (obese). This parameter was chosen because it is considered one of the most reliable indicators of animal health, productive performance, and reproductive status, as reported by Roche and colleagues [54].

Statistical analysis of blood parameters and gastrointestinal strongyles was performed using ANOVA. Data regarding FECs were logarithmically transformed [y = log (EPG + 25)] to normalize error [55]. Statistical analyses were performed using GraphPad Prism 9 (Graph-Pad Software, San Diego, CA, USA). Statistical significance was set at *p* ≤ 0.05. All the data are also provided in the Supplementary Materials (Table S1).

## 3. Results

Mean EPG values (±S.E.), calculated across the three sampling time points, were 210 ± 78.10 for group T and 529.05 ± 88.92 for group NT, and differed significantly between the two groups (t = 3.55; *p* = 0.0007).

**Figure 2:**
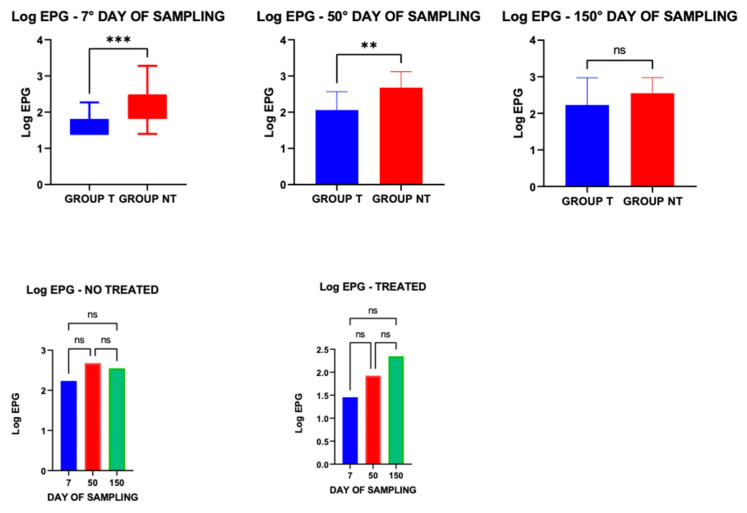
Comparison of the levels of parasitic infestation (log EPG) between animals of the treated (T) and non-treated (NT) groups in the three samplings. *** *p* ≤ 0.001; ** *p* ≤ 0.01; ns = not significant.

EPG values in the T group were significantly lower than those in the NT group at 7 days post-treatment. However, no significant difference was observed at 150 days. Furthermore, intra-group comparisons across the three time points revealed no statistically significant differences, likely due to high inter-individual variability (Figure 2).

Table 1 summarizes the median EPG values and the corresponding minimum–maximum ranges recorded at each sampling time point. Although a reduction in EPG was observed 7 days after treatment, marked inter-individual variability was evident.

The distribution of EPG levels in the two groups is reported in Table 2. Animals in group NT exhibited a higher level of infestation, particularly at EPG level 2. Moreover, a substantial proportion of animals in group NT showed level 3 infestation.

No cestode proglottids were observed on macroscopic examination. Coproculture revealed a predominance of *Trichostrongylus* spp. larvae in the examined fecal samples, while *Chabertia ovina* was detected at a lower frequency.

Regarding BCS, the mean values (±S.E.) calculated across the three sampling time points for the two groups were 2.51 ± 0.53 in T group and 3.00 ± 0.61 in NT group; a significant difference was observed between groups (t = 3.548, *p* = 0.0007), with animals in group NT exhibiting a better body condition.

**Figure 3:**
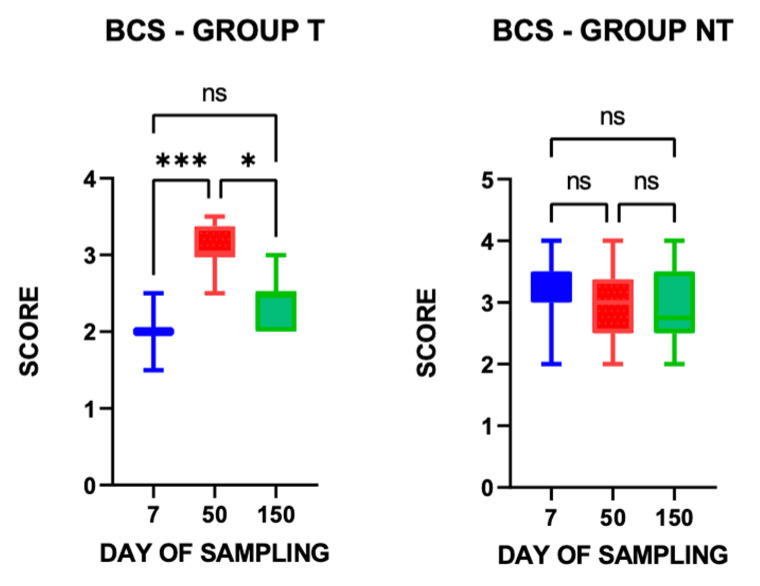
Relative Body Condition Score (BCS) values in treated (T) and non-treated (NT) groups at the three sampling time points. * *p* ≤ 0.05; *** *p* ≤ 0.001; ns = not significant.

BCS values remained stable in animals from group NT across the three sampling time points. In contrast, a statistically significant reduction in BCS was observed in group T at 7 and 150 days post-treatment (Figure 3).

The BCS mean value (±S.E.) of animals of group T (2.05 ± 0.09), assessed 7 days after the second anthelmintic treatment, was significantly lower than that of animals in group NT (3.25 ± 0.17) (*p* ≤ 0.0001).

During the study period, none of the animals in group NT exhibited a reduction in BCS sufficient to require veterinary evaluation or the administration of anthelmintic treatment.

Table 3 reports the mean hair cortisol concentrations (±S.E.) according to EPG level. The highest mean value was observed at EPG level 3; however, no statistically significant differences were detected among the three levels.

**Figure 4:**
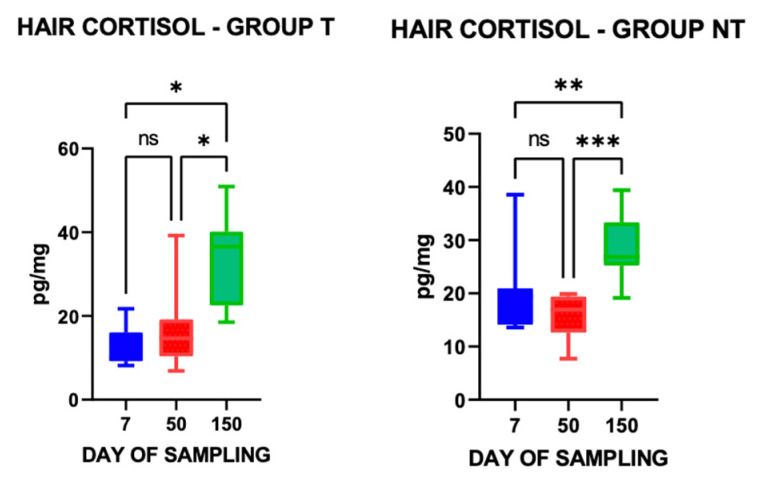
Hair cortisol concentrations (pg/mL) in treated (T) and non-treated (NT) groups at the three sampling time points. * *p* ≤ 0.05; ** *p* ≤ 0.01; *** *p* ≤ 0.001; ns = not significant.

When comparing hair cortisol concentrations across the three sampling time points, values measured 150 days after treatment were significantly higher in both groups T and NT than those recorded at 7 and 50 days post-treatment (Figure 4).

A statistically significant difference in mean (±S.E.) hair cortisol concentrations was also observed between groups T (9.97±), and NT (20.08 ± 2.09), at 7 days post-treatment (U = 20; *p* ≤ 0.05).

Analysis of the haematochemical parameters reported in Table 4 reveal no statistically significant differences between groups. Although not statistically significant, mean RBC and HCT values in group T were slightly below the physiological reference range.

Table 5 reports the mean values of metabolic parameters of animals of the T and NT group.

**Figure 5:**
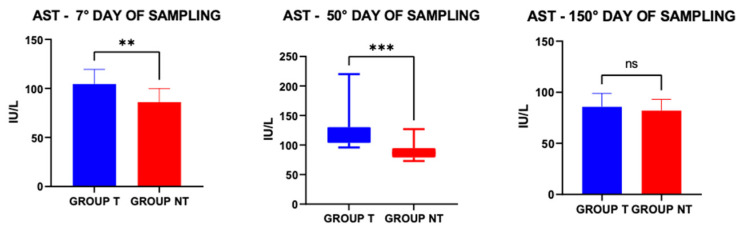
Serum Aspartate Aminotransferase (AST) concentrations in treated (T) and non-treated (NT) groups at the three different samplings. ** *p* ≤ 0.01; *** *p* ≤ 0.001; ns = not significant.

ALT mean values (±S.E.) at 7 (25.60 IU ± 1.4) and 50 (24.55 IU ± 1.40) days are higher than those detected 150 days after deworming (18.00 ± 1.13). A repeated-measures one-way ANOVA showed a significant effect of sampling time on ALT values [F (1.56, 12.48) = 6.78, *p* = 0.0139]. Tukey’s multiple comparisons test indicated that ALT values at 150 days were significantly lower than those measured at 7 days (*p* = 0.0327) and 50 days (*p* = 0.0063), while no significant difference was observed between 7 and 50 days (*p* = 0.9723). Mean AST values differed significantly between groups (U = 286.5; *p* = 0.0003); moreover, AST concentrations in group T animals were elevated at 7 and 50 days, with a statistically significant difference compared to those detected 150 days after treatment, although remaining within the range considered physiological (Figure 5).

**Figure 6:**
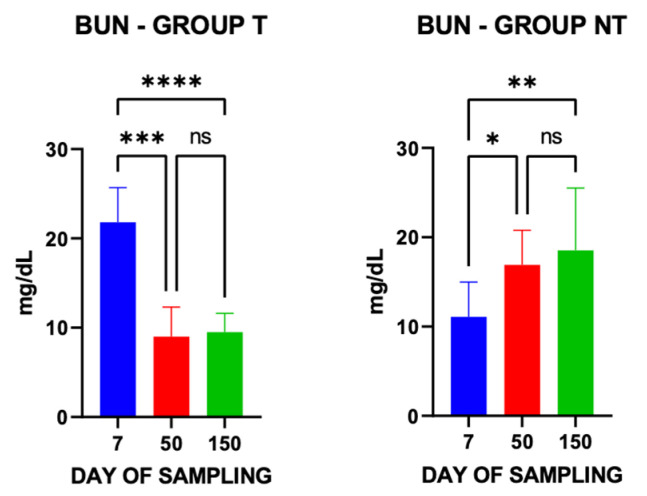
Blood Urea Nitrogen (BUN) concentrations in treated (T) and non-treated (NT) groups at the three sampling time points. * *p* ≤ 0.05; ** *p* ≤ 0.01; *** *p* ≤ 0.001; **** *p* ≤ 0.0001; ns = not significant.

BUN concentrations in group T were significantly higher at 7 days post-treatment compared with those measured at 50 and 150 days after deworming, whereas an opposite trend was observed in group NT (Figure 6).

Analysis of potential correlations among cortisol levels (in blood and hair), BCS, EPG, and biochemical and haematological parameters revealed the significant associations reported in Table 6.

## 4. Discussion

The expansion of land use and the increasing prevalence of intensive livestock farming require a comprehensive evaluation of the sustainability of animal production systems, a concept that has broadened considerably compared with earlier interpretations. Traditionally, production systems were considered unsustainable when natural resources were depleted beyond their capacity for recovery or when by-products accumulated to levels that impaired system functioning [56]. The concept now encompasses a broader scope; for example, a production system may be considered unsustainable because of its negative impacts on human health, animal welfare, or the environment. Moreover, consumers increasingly take the ethical implications of food production into account when evaluating product quality [57]. A system or procedure can be defined as sustainable if it is acceptable under current conditions and if its projected future impacts are likewise acceptable, taking into account resource availability, operational performance, and ethical considerations [58,59]. Livestock farming poses substantial challenges to environmental sustainability, even under extensive management systems. Key concerns include animal health and welfare, pasture overgrazing, the spread of drug-resistant parasites, and residues of antiparasitic compounds in the environment. This study compared two parasite control strategies in sheep in order to evaluate the feasibility of environmentally sustainable flock management without routine pesticide use, while maintaining adequate standards of animal health and welfare. Analysis of the factors influencing animal welfare indicated marked inter-individual variability in parasite load and a limited duration of the antiparasitic treatment effect. In fact, 150 days after treatment, no differences were observed between animals in group T and those in group NT. Additionally, treated animals exhibited a negative correlation between EPG and MCV at 7 days post-treatment, although MCV values remained within the physiological range for the species. This transient effect highlights the need for repeated treatments, which in turn raises concerns regarding the development of anthelmintic resistance [21]. Recent studies have shown that the overuse of broad-spectrum anthelmintics can accelerate resistance even in small, closed flocks [60]. From a sustainability perspective, reliance on chemical parasite control not only compromises the long-term efficacy of treatments but also generates environmental and economic challenges for livestock production. By contrast, group NT maintained relatively stable and moderate EPG levels in the absence of pharmacological intervention. Although correlation does not necessarily imply causation, in this group EPG was significantly and positively correlated with blood cortisol concentrations and reticulocyte counts, suggesting a physiological stress response associated with increasing parasite burden. This interpretation is further supported by the observed negative correlations between EPG and HGB, NEFA, and Ca. Previous studies [61,62] have demonstrated that parasitic infestations can influence key hematological parameters; in particular, subclinical anaemia may occur as a consequence of micro-hemorrhages caused by nematode attachment and larval migration, and a slight reduction in HGB concentration may represent an early and subtle indicator of parasitic challenge. These findings represent a warning signal that deserves careful monitoring in flocks managed without routine anthelmintic treatment.

Coproculture of pooled fecal samples identified *Chabertia ovina* and *Trichostrongylus* spp. as the main gastrointestinal nematodes present in the monitored sheep flocks. The two parasites differ in their ecological dynamics, with *Trichostrongylus* spp. showing rapid development and early pasture contamination under favorable environmental conditions, whereas *Chabertia ovina* exhibits slower larval development but greater environmental persistence once established on pasture [63]. In both cases, larval availability on herbage is strongly moisture-dependent, with peak exposure following rainfall or heavy dew [64]. Despite their presence, EPG values remained quite low, and no severe clinical signs were observed, in line with the typically mild to moderate pathogenicity associated with these nematodes under conditions of low parasite burden [65]. The absence of hematophagous species such as *Haemonchus contortus* further supports the limited clinical impact recorded in the studied flocks.

Another important factor in the assessment of animal welfare is body condition score (BCS). Animals in group NT exhibited a significantly higher mean BCS than those in group T and the values remained stable in the NT flock throughout the study period, whereas a statistically significant reduction in BCS was observed in group T at 7 and 150 days post-treatment. In addition, the BCS of treated animals assessed 7 days after the second anthelmintic treatment was significantly lower than that of animals in group NT.

Resilience represents a key adaptive trait in sustainable livestock farming, particularly under conditions of parasitic challenge [66,67]. Moreover, in extensive production systems, BCS may be influenced more strongly by environmental, nutritional, and behavioral factors than by parasite burden alone, as noted by Kenyon and Jackson [68]. However, in the present study, these potential confounding factors can be reasonably excluded, as environmental and nutritional conditions, as well as management practices, were comparable between the two farms.

Haematological analyses revealed only mild differences between the two groups. Although RBC, HGB, and HCT values were slightly below the reference ranges in both flocks, these variations were not statistically significant and may reflect physiological adaptation to chronic parasitism. The reduced RBC, HCT, and HGB levels observed are consistent with the findings of Aziz et al. [69], who reported similar decreases associated with intestinal parasitic infections.

Thrombocytopenia in ruminants may be associated with blood loss, septicemia and inflammatory diseases such as mastitis and metritis [70]. However, in the present study, the lower platelet counts observed in group NT compared with group T were not associated with any clinical signs consistent with these conditions [71].

Serum biochemical analysis revealed notable differences in markers related to liver and kidney function, as well as energy metabolism. ALT values exceeded the physiological reference range in both experimental groups, although no significant differences were detected between groups. In sheep, ALT is not considered a specific marker of hepatic injury, unlike in dogs or humans; nevertheless, increased ALT activity has been reported following the administration of anthelmintic drugs such as albendazole, which undergoes hepatic metabolism. This interpretation is supported by the observation that ALT values exceeded the physiological range at 7 and 50 days after deworming.

By contrast, AST is regarded as a more sensitive indicator for the diagnosis of fatty liver in sheep [72]. This enzyme is localized in both the cytoplasm and mitochondria of various tissues, with the highest activity found in skeletal muscle, heart, and liver. Consequently, alterations in circulating AST activity may reflect damage to cellular structures, particularly within hepatic tissue [73]. AST concentrations were significantly higher in animals from group T, pointing to a greater degree of liver involvement, potentially associated with pharmacological metabolism. In fact, albendazole undergoes hepatic metabolism following oral administration through a sequential two-step sulphoxidation pathway [74]. This process initially produces albendazole sulphoxide via a rapid and reversible reaction, followed by further oxidation mediated by cytochrome P450 (CYP) enzymes, resulting in the inactive metabolite albendazole sulphone [75,76]. These CYP-driven reactions are accompanied by the generation of reactive oxygen and nitrogen species (ROS and RNS), and the transient release of reactive intermediates from the metabolic system has been documented [77]. Such biochemical events may underlie the elevated AST activity observed in treated animals.

In group T, BUN levels 7 days after treatment were statistically higher than those of the other 2 samples: one cause could be a reduction in renal blood flow due to dehydration resulting from diarrhea, a possible side effect of treatment with albendazole [78].

NEFA concentrations were below the physiological reference range in both groups and were more markedly reduced in group T. In sheep with chronic gastrointestinal nematode infections, alterations of energy metabolism have been reported, including normal or reduced circulating NEFA concentrations, likely reflecting reduced lipid mobilization and energy reallocation towards immune responses rather than acute negative energy balance [79].

Oxidative stress, assessed through OFR, was higher in group NT, although the difference did not reach statistical significance. Oxidative stress is a multifactorial phenomenon and should not be attributed solely to pharmacological treatment. Additional contributing factors may include chronic parasitic burden, nutritional imbalances, environmental stressors, as well as physiological or social influences [80].

With respect to hair cortisol, although the highest concentrations were observed in animals with EPG level 3, no statistically significant differences were detected among EPG categories. Likewise, hair cortisol concentrations did not differ significantly between groups T and NT (18.33 vs. 16.67 pg/mg), indicating comparable long-term cortisol levels: this finding could reflect an inherent resilience trait in the animals. However, when animals were stratified by EPG levels, hair cortisol increased with parasite load; moreover, hair cortisol levels were slightly higher in sheep with high EPG (25.01 pg/mg) compared to those with low EPG (22.30 pg/mg), but the difference was not statistically significant. These findings are consistent with those of Carlsson et al. [81], who observed that although cortisol levels may rise with parasitic burden, they are not reliable as a standalone indicator of parasite infection.

## 5. Conclusions

This study highlights the multifaceted effects of different gastrointestinal parasite management strategies on sheep health. Although the findings should be considered preliminary, given the limited number of animals, the short duration of the study, and the inclusion of a particularly resilient sheep breed, the results appear to support the sustainability of sheep farming systems that do not rely on routine anthelmintic treatments, both in terms of animal welfare and environmental impact.

Although the absence of anthelmintic treatment may expose animals to potential health and production losses associated with parasitic infection, careful and continuous monitoring, enabling timely intervention in response to declines in BCS or alterations in haematological and biochemical parameters, can effectively support animal health and welfare.

On the other hand, the use of anthelmintic drugs is not without risk. In addition to the environmental concerns associated with drug residues, anthelmintic administration may act as a stressor for animals, particularly in the period immediately following treatment. In the present study, animals evaluated 7 days post-treatment showed a reduction in BCS, increased BUN concentrations, elevated AST activity, and ALT values exceeding the physiological reference range.

Despite the absence of pharmacological treatment, animals in group NT exhibited higher BCS values and showed no significant indications of chronic stress, as reflected by hair cortisol concentrations. Overall, these findings suggest that the adoption of integrated, low-chemical parasite control strategies may enhance the sustainability of livestock systems by reducing reliance on drugs, limiting environmental contamination, and preserving the long-term efficacy of available anthelmintics. Promoting resilience within such systems supports both animal welfare and productivity, ensuring that parasite management is aligned with the broader environmental, economic, and ethical objectives of modern sheep farming. The results obtained are therefore relevant and may provide a basis for extending this approach to other sheep breeds and diverse climatic conditions.

## Figures and Tables

**Table 1:** Median EPG values and corresponding minimum–maximum ranges recorded in the non-treated group (NT) and treated group (T) at each sampling time point.

Group	Sampling	Days from Drenching	Median EPG	Min–Max
T	1	50	140.0	0–920
T	2	7	0.0	0–160
T	3	150	40.0	0–1680
NT	1	-	300.0	80–1680
NT	2	-	440.0	40–1680
NT	3	-	200.0	0–1880

**Table 2:** Distribution of EPG levels in the treated (T) and non-treated (NT) groups.

	Group	
	T	NT
EPG Level	% of Sampled Animals	
1	80.9	37.50
2	0	33.33
3	19.0	29.17

**Table 3:** Mean hair cortisol concentrations (±S.E.) according to Eggs Per Gram (EPG) level.

	Hair Cortisol	
EPG Level	Mean (pg/mg)	S.E.
1	22.30	1.793
2	19.09	4.009
3	25.01	3.472

**Table 4:** Haematochemical parameters in the treated (T) and non-treated (NT) groups.

	T		NT				
	Mean	S.E.	Mean	S.E.	*p*	UM	Normal Range *
Red Blood Cells	8.79 ↓	0.35	8.32 ↓	0.35	0.347	M/µL	9.49–15.12
HCT	26.83 ↓	1.15	26.24 ↓	1.15	0.717	%	27.0–42.0
HGB	9.69 ↓	0.45	9.02 ↓	0.45	0.297	g/dL	10.0–14.9
MCV	30.56	0.69	31.61	0.69	0.285	fl	24.4–32.5
MCH	11.10	0.38	10.88	0.38	0.692	pg	8.5–11.8
MCHC	36.49	1.29	34.44	0.29	0.268	g/dL	32.3–42.0
Reticulocytes	3.07	1.81	3.98	1.81	0.722	K/µL	0–15.0
Leucocytes	7.92	0.66	7.98	0.66	0.949	K/µL	5.06–14.12
Neutrophyls	2.08	0.28	2.63	0.28	0.171	K/µL	1.17–6.11
Lymphocytes	4.08	0.34	3.79	0.34	0.531	K/µL	2.54–9.60
Monocytes	1.00	0.15	0.86 ↓	0.14	0.483	K/µL	0.10–1.01
Eosinophils	0.68	0.17	0.62	0.17	0.805	K/µL	0.05–0.95
Basophils	0.14 ↑	0.03	0.10	0.03	0.373	K/µL	0–0.12
Platelets	447.25	62.10	265.83 ↓	62.10	0.042	K/µL	301–922
MPV	9.78	0.28	9.04	0.28	0.068	fl	5.0–7.5

HCT (haematocrit), HGB (haemoglobin), MCV (mean corpuscular volume), MCH (mean corpuscular haemoglobin), MCHC (mean corpuscular haemoglobin concentration), and MPV (Mean Platelet Volume). * Reference intervals were provided by the Veterinary Clinical Pathology Laboratory, Department of Veterinary Sciences, University of Pisa. ↑ Values over the threshold of the normal range; ↓ Values under the threshold of the normal range.

**Table 5:** Mean values (±S.E.) of metabolic parameters in the treated (T) and non-treated (NT) groups.

	Group						
	T		NT				
	Mean	S.E.	Mean	S.E.	*p*	UM	Normal Range *
ALT	24.54 ↑	1.521	23.75 ↑	1.456	0.7068	IU/L	≤18
AST	123.00	5.655	90.50	5.414	0.0001	IU/L	40–128
BUN	9.27	1.306	16.58	1.251	0.0001	mg/dL	8–20
BHBA	7.83	1.044	6.04	0.999	0.2203	mmol/L	5.7–20
Creatinine	0.81	0.029	0.80	0.027	0.7415	mg/dL	1.2–1.9
Cortisol	1.91	0.18	2.38	0.20	0.0859	mg/dL	-
NEFA	91.09 ↓	70.047	136.64	67.065	0.6403	μmol/L	102–450
TP	7.08	0.192	7.14	0.184	0.8229	g/dL	6–7.90
Ca	9.72 ↓	0.174	9.78 ↓	0.167	0.8147	mg/dL	11.5–13
Cl	106.82 ↑	0.698	108.33	0.668	0.1221	Mmol/L	90–110
P	4.45	0.259	4.07	0.248	0.2919	mg/dL	4.5–7.5
K	4.58 ↓	0.153	4.70 ↓	0.146	0.5790	mmol/L	4.8–7
OFR	38.45	4.989	48.92	4.777	0.1350	U. Carr.	44–88
Hair cortisol	18.33	2.608	16.67	2.497	0.6470	pg/mg	-

ALT (alanine aminotransferase), AST (aspartate aminotransferase), BUN (blood urea nitrogen), BHBA (β--hydroxybutyric acid), NEFA (non-esterified fatty acids), TP (total proteins), Ca (calcium), Cl (chloride), P (phosphorus) and K (potassium), OFR (Oxygen free radicals), Cortisol (serum cortisol). * Reference ranges were provided by the IZSLT laboratory. U. Carr. is an arbitrary unit; 1 U. Carr. is equivalent to 0.08 mg of H_2_O_2_/100 mL. ↑ Values over the threshold of the normal range; ↓ Values under the threshold of the normal range.

**Table 6:** Significant correlations among Eggs Per Gram (EPG), Body Condition Score (BCS), haematological, biochemical, and hormonal parameters in treated (T) and non-treated (NT) groups.

Animal Group	Parameters	Type of Correlation	Days After Treatment	Rho
T	EPG-MCV	Negative	7	−0.68; *p* = 0.04
T	Hair cortisol-BCS	Negative	7	−0.69; *p* = 0.02
T	Hair Cortisol-Ca	Negative	7	−0.77; *p* = 0.01
T	Hair cortisol-BCS	Negative	50	−0.65; *p* = 0.02
T	Hair cortisol-P	Negative	50	−0.65; *p* = 0.03
T	HGB–Cortisol	Negative	150	−0.79; *p* = 0.003
NT	Hair Cortisol-Ca	Negative	7	−0.69; *p* = 0.03
NT	BCS-HCT	Positive	7	0.85; *p* ≤ 0.001
NT	BCS-HGB	Positive	7	0.77; *p* = 0.005
NT	BCS-Ca	Positive	7	0.62; *p* = 0.04
NT	EPG-HGB	Negative	7	−0.63; *p* = 0.03
NT	EPG-Reticulocytes	Positive	50	0.60; *p* = 0.04
NT	EPG-Cortisol	Positive	50	0.62; *p* = 0.04
NT	EPG-NEFA	Negative	50	−0.64; *p* = 0.03
NT	EPG-Ca	Negative	150	−0.59; *p* = 0.05

EPG = Eggs Per gram; MCV = mean corpuscular volume; BCS = Body Condition Score; Ca = Calcium; P = Phosphorus; HGB = Haemoglobin; Cortisol = serum cortisol; NEFA = Non-Esterified Fatty Acids.

## Data Availability

The original contributions presented in this study are included in the article. Further inquiries can be directed to the corresponding author.

## Supplementary Materials

The following supporting information can be downloaded at https://www.mdpi.com/article/10.3390/vetsci13010104/s1. Table S1: Data sets.

## References

[1] Maurizio A., Perrucci S., Tamponi C., Scala A., Cassini R., Rinaldi L., Bosco A. (2023). Control of Gastrointestinal Helminths in Small Ruminants to Prevent Anthelmintic Resistance: The Italian Experience. Parasitology.

[2] Charlier J., Bartley D.J., Sotiraki S., Martinez-Valladares M., Claerebout E., von Samson-Himmelstjerna G., Thamsborg S.M., Hoste H., Morgan E.R., Rinaldi L. (2022). Anthelmintic Resistance in Ruminants: Challenges and Solutions. Adv. Parasitol..

[3] Jackson F., Bartley D., Bartley Y., Kenyon F. (2009). Worm Control in Sheep in the Future. Small Rumin. Res..

[4] Houdijk J.G.M., Tolkamp B.J., Rooke J.A., Hutchings M.R. (2017). Animal Health and Greenhouse Gas Intensity: The Paradox of Periparturient Parasitism. Int. J. Parasitol..

[5] Louie K., Vlassoff A., Mackay A.D. (2007). Gastrointestinal Nematode Parasites of Sheep: A Dynamic Model for Their Effect on Liveweight Gain. Int. J. Parasitol..

[6] Hoste H., Meza-Ocampos G., Marchand S., Sotiraki S., Sarasti K., Blomstrand B.M., Williams A.R., Thamsborg S.M., Athanasiadou S., Enemark H.L. (2022). Use of Agro-Industrial by-Products Containing Tannins for the Integrated Control of Gastrointestinal Nematodes in Ruminants. Parasite.

[7] Kenyon F., Greer A.W., Coles G.C., Cringoli G., Papadopoulos E., Cabaret J., Jackson F. (2009). The Role of Targeted Selective Treatments in the Development of Refugia-Based Approaches to the Control of Gastrointestinal Nematodes of Small Ruminants. Vet. Parasitol..

[8] Köhler P. (2001). The Biochemical Basis of Anthelmintic Action and Resistance. Int. J. Parasitol..

[9] Vokřála I., Jirásko R., Stuchlíková L., Bártíková H., Szotáková B., Lamka J., Várady M., Skálová L. (2013). Biotransformation of Albendazole and Activities of Selected Detoxification Enzymes in *Haemonchus contortus* Strains Susceptible and Resistant to Anthelmintics. Vet. Parasitol..

[10] Hamscher G., Bachour G. (2018). Veterinary Drugs in the Environment: Current Knowledge and Challenges for the Future. J. Agric. Food Chem..

[11] Marsík P., Podlipná R., Vaněk T. (2017). Study of Praziquantel Phytoremediation and Transformation and Its Removal in Constructed Wetland. J. Hazard. Mater..

[12] Stuchlíková Raisová L., Podlipná R., Szotáková B., Syslová E., Skálová L. (2017). Evaluation of Drug Uptake and Deactivation in Plant: Fate of Albendazole in Ribwort Plantain (*Plantago lanceolata*) Cells and Regenerants. Ecotoxicol. Environ. Saf..

[13] Bártíková H., Podlipná R., Skálová L. (2016). Veterinary drugs in the environment and their toxicity to plants. Chemosphere.

[14] Syslová E., Landa P., Navrátilová M., Stuchlíková Raisová L., Matoušková P., Skálová L., Szotáková B., Vaněk T., Podlipná R. (2019). Ivermectin biotransformation and impact on transcriptome in *Arabidopsis thaliana*. Chemosphere.

[15] Syslová E., Landa P., Stuchlíková Raisová L., Matoušková P., Skálová L., Szotáková B., Navrátilová M., Vaněk T., Podlipná R. (2019). Metabolism of the anthelmintic drug fenbendazole in *Arabidopsis thaliana* and its effect on transcriptome and proteome. Chemosphere.

[16] Belew S., Suleman S., Wynendaele E., Duchateau L., De Spiegeleer B. (2021). Environmental risk assessment of the anthelmintic albendazole in Eastern Africa, based on a systematic review. Environ. Pollut..

[17] Jensen J., Durand M., Permin A., Roepstorff A. (2009). Single- and two species tests to study effects of the anthelmintics ivermectin and morantel and the coccidiostatic monensin on soil invertebrates. Environ. Toxicol. Chem..

[18] Jensen J., Lambkin T.M., Roberts D.W. (2003). Effects of the antibacterial agents tiamulin, olaquindox and metronidazole and the anthelmintic ivermectin on the soil invertebrate species *Folsomia fimetaria* (Collembola) and *Enchytraeus crypticus* (Enchytraeidae). Chemosphere.

[19] Puckowski A., Stolte S., Wagil M., Markiewicz M., Łukaszewicz P., Stepnowski P., Białk-Bielińska A. (2017). Mixture toxicity of flubendazole and fenbendazole to *Daphnia magna*. Int. J. Hyg. Environ. Health.

[20] Ng’etich A.I., Amoah I.D., Bux F., Kumari S. (2024). Anthelmintic Resistance in Soil transmitted Helminths: One Health Considerations. Parasitol. Res..

[21] Kaplan R.M. (2004). Drug Resistance in Nematodes of Veterinary Importance: A Status Report. Trends Parasitol..

[22] Sutherland I.A., Leathwick D.M. (2011). Anthelmintic Resistance in Nematode Parasites of Cattle: A Global Issue?. Trends Parasitol..

[23] Abbott K., Taylor M.A. (2012). Sustainable Worm Control Strategies for Sheep. A Technical Manual for Veterinary Surgeons and Advisors.

[24] Doyle S.R., Laing R., Bartley D., Morrison A., Holroyd N., Maitland K., Antonopoulos A., Chaudhry U., Flis I., Howell S. (2022). Genomic Landscape of Drug Response Reveals Mediators of Anthelmintic Resistance. Cell Rep..

[25] Hassan N.M.F., Ghazy A.A. (2021). Advances in Diagnosis and Control of Anthelmintic Resistant Gastrointestinal Helminths Infecting Ruminants. J. Parasit. Dis..

[26] Vokřál I., Podlipná R., Matoušková P., Skálová L. (2023). Anthelmintics in the Environment: Their Occurrence, Fate, and Toxicity to Non target Organisms. Chemosphere.

[27] Burke J. (2020). Sustainable Approaches to Parasite Control in Ruminant Livestock. Vet. Clin. N. Am. Food Anim. Pract..

[28] Hoste H., Jackson F., Athanasiadou S., Thamsborg S.M., Hoskin S.O. (2006). The Effects of Tannin-Rich Plants on Parasitic Nematodes in Ruminants. Trends Parasitol..

[29] Maqbool I., Wani Z.A., Shahardar R.A., Allaie I.M., Shah M.M. (2017). Integrated Parasite Management with Special Reference to Gastro-Intestinal Nematodes. J. Parasit. Dis..

[30] Burke J.M., Miller J.E., Terrill T.H. (2007). Use of Copper Oxide Wire Particles to Control Gastrointestinal Nematodes in Goats. J. Anim. Sci..

[31] Larsen M. (1999). Biological Control of Gastro-Intestinal Nematodes of Ruminants: Expert Consultation.

[32] Rodriguez-Hernández P., Reyes-Palomo C., Sanz-Fernández S., Rufino-Moya P.J., Zafra R., Martinez-Moreno F.J., Rodriguez-Estévez V., Díaz-Gaona C. (2023). Antiparasitic Tannin-Rich Plants from the South of Europe for Grazing Livestock: A Review. Animals.

[33] Quadros D., Burke J. (2024). Nutritional Strategies and Integrated Control of Gastrointestinal Nematodes in Small Ruminants. Anim. Front..

[34] Mendoza-de Gives P., López-Arellano M.E., Olmedo-Juárez A., Higuera-Pierdrahita R.I., von Son-de Fernex E. (2023). Recent Advances in the Control of Endoparasites in Small Ruminants. Pathogens.

[35] Colvin A.F., Walkden Brown S.W., Knox M.R., Scott J.M. (2008). Intensive Rotational Grazing Assists Control of Gastrointestinal Nematodosis of Sheep in a Cool Temperate Environment with Summer dominant Rainfall. Vet. Parasitol..

[36] Marley C.L., Fraser M.D., Davies D.A., Rees M.E., Vale J.E., Forbes A.B. (2006). The Effect of Mixed and Sequential Grazing of Cattle and Sheep on the Faecal Egg Counts and Growth Rates of Weaned Lambs When Treated with Anthelmintics. Vet. Parasitol..

[37] Malik M.A., Amin A.B., Iqbal Z., Bajwa M.R.K., Aleem M.T., Mansoor M.A., Timpong Jones E.C. (2023). Parasite Control Strategies: Pasture Management. Parasitism and Parasitic Control in Animals: Strategies for the Developing World.

[38] Kumar D., Swarnkar C.P., Singh D., Khan F.A. (2012). Internal Parasite Management in Grazing Livestock. Vet. Pract..

[39] Van Wyk J.A., Hoste H., Kaplan R.M., Besier R.B. (2006). Targeted Selective Treatment for Worm Management—How Do We Sell Rational Programs to Farmers?. Vet. Parasitol..

[40] Hodgkinson J.E., Kaplan R.M., Kenyon F., Morgan E.R., Park A.W., Paterson S., Devaney E. (2019). Refugia and Anthelmintic Resistance: Concepts and Challenges. Int. J. Parasitol. Drugs Drug Resist..

[41] Albers G.A., Gray G.D., Piper L.R., Barker J.S., Le Jambre L.F., Barger I.A. (1987). The Genetics of Resistance and Resilience to Haemonchus Contortus Infection in Young Merino Sheep. Int. J. Parasitol..

[42] Bishop S.C. (2015). Genetic Resistance to Infections in Sheep. Vet. Microbiol..

[43] Karrow N.A., Goliboski K., Stonos N., Schenkel F., Peregrine A. (2014). Review: Genetics of Helminth Resistance in Sheep. Can. J. Anim. Sci..

[44] Kumar S., Kumar M., Yadav D., Kumar B., Kumar Singh R. (2024). Integrated Parasite Management in Small Ruminants. Indian. Farmer..

[45] O’Connor L.J., Walkden-Brown S.W., Kahn L.P. (2006). Grazing Management and Helminth Control on Stock-Grazed Pastures: A Review. Vet. Parasitol..

[46] Permin A., Hansen J. (1998). Epidemiology, Diagnosis and Control of Poultry Parasites.

[47] Ambrosi M. (1995). Parassitologia Zootecnica.

[48] Molento M.B., Gaviao A.A., Depner R.A., Pires C.C. (2016). Pasture Larval Counts as a Supporting Method for Parasite Risk Assessment. Vet. Parasitol..

[49] Henriksen S.A., Korsholm H. (1983). A Method for Culture and Recovery of Gastrointestinal Strongyle Larvae. Nord. Vet. Med..

[50] Taylor M.A. (2012). Emerging Parasitic Diseases of Sheep. Vet. Parasitol..

[51] Mariti C., Diverio S., Gutiérrez J., Baragli P., Gazzano A. (2020). Partial Analytic Validation of Determination of Cortisol in Dog Hair Using a Commercial EIA Kit. Dog Behav..

[52] Gazzano V., Curadi M.C., Baragli P., Mariti C., Cecchi F., Cavallo S., Sacchettino L., Gazzano A. (2025). Physiological and Behavioral Evaluation of Shelter Dogs During Veterinary Routine Health Checks. Vet. Sci..

[53] Russel A.J.F. (1984). Means of Assessing the Adequacy of Nutrition in Ewes. Livest. Prod. Sci..

[54] Roche J.R., Friggens N.C., Kay J.K., Fisher M.W., Stafford K.J., Berry D.P. (2009). Invited Review: Body Condition Score and Its Association with Health, Milk Production, Wasting, and Fertility. J. Dairy. Sci..

[55] Baker R.L. (1997). Résistance Génétique Des Petits Ruminants Aux Helminthes En Afrique. INRA Prod. Anim..

[56] Broom D.M. (2002). Does Present Legislation Help Animal Welfare?. Landbauforsch. Völkenrode.

[57] Broom D.M. (2010). Animal Welfare: An Aspect of Care, Sustainability, and Food Quality Required by the Public. J. Vet. Med. Educ..

[58] Broom D.M. (2014). Sentience and Animal Welfare.

[59] Broom D.M. (2017). Components of Sustainable Animal Production and the Use of Silvopastoral Systems. Rev. Bras. Zootec..

[60] Vineer H.R., Morgan E.R., Hertzberg H., Bartley D.J., Bosco A., Charlier J., Rinaldi L. (2020). Increasing Importance of Anthelmintic Resistance in European Livestock: Creation and Meta-Analysis of an Open Database. Parasite.

[61] Kumar S., Jakhar K.K., Singh S., Potliya S., Kumar K., Pal M. (2015). Clinicopathological Studies of Gastrointestinal Tract Disorders in Sheep with Parasitic Infection. Vet. World.

[62] Awad A.H., Ali A.M., Hadree D.H. (2016). Some Haematological and Biochemical Parameters Assessments in Sheep Infection by Haemonchus Contortus. Tikrit J. Pure Sci..

[63] Gyeltshen T., Kahn L.P., Laurenson Y.C.S.M. (2022). Ecology of the free-living stages of Trichostrongylid parasites of sheep. Vet. Parasitol..

[64] Bricarello P.A., Longo C., da Rocha R.A., Hötzel M.J. (2023). Understanding Animal–Plant–Parasite Interactions to Improve the Management of Gastrointestinal Nematodes in Grazing Ruminants. Pathogens.

[65] Knoll S., Dessì G., Tamponi C., Meloni L., Cavallo L., Mehmood N., Jacquiet P., Scala A., Cappai M.G., Varcasia A. (2021). Practical Guide for Microscopic Identification of Infectious Gastrointestinal Nematode Larvae in Sheep from Sardinia, Italy. Parasit Vectors.

[66] Cunha S.M.F., Willoughby O., Schenkel F., Cánovas Á. (2024). Genetic Parameter Estimation and Selection for Resistance to Gastrointestinal Nematode Parasites in Sheep—A Review. Animals.

[67] Jackson F., Coop R.L., Jackson E. (2009). The Role of Targeted Selective Treatments in the Development of Anthelmintic Resistance in Sheep. N. Z. Vet. J..

[68] Kenyon F., Jackson F. (2012). Targeted Flock/Herd and Individual Ruminant Treatment Approaches. Vet. Parasitol..

[69] Abd El-Aziz M., Kassem J.M., Aasem F.M., Abbas H.M. (2022). Physicochemical Properties and Health Benefits of Camel Milk and Its Applications in Dairy Products: A Review. Egypt. J. Chem..

[70] Stockham S.L., Scott M.A. (2008). Fundamentals of Veterinary Clinical Pathology.

[71] Polizopoulou Z.S. (2010). Haematological Tests in Sheep Health Management. Small Rumin. Res..

[72] Constable P.D., Hinchcliff K.W., Done S.H. (2017). Veterinary Medicine: A Text Book of the Disease of Cattle, Horse, Sheep, Pigs and Goats.

[73] Topchiyeva S.A. (2018). Change in the Enzymatic Activity of Aspartate Aminotransferase in the Blood of Goats Related to the State of Animal Health. J. Med. Res. Biol. Stud..

[74] Velik J., Baliharova V., Fink-Gremmels J., Bull S., Lamka J., Skalova L. (2004). Benzimidazole Drugs and Modulation of Biotransformation Enzymes. Res. Vet. Sci..

[75] Skalova L., Krizova V., Cvilink V., Szotakova B., Storkanová L., Velik J., Lamka J. (2007). Mouflon (*Ovis musimon*) Dicrocoeliosis: Effects of Parasitosis on the Activities of Biotransformation Enzymes and Albendazole Metabolism in Liver. Vet. Parasitol..

[76] Capece B.P.S., Afonso S.M.S., Lazaro R., Harun M., Godoy C., Castells G., Cristofol C. (2009). Effect of Age and Gender on the Pharmacokinetics of Albendazole and Albendazole Sulphoxide Enantiomers in Goats. Res. Vet. Sci..

[77] Guengerich F.P. (2008). Cytochrome P450 and Chemical Toxicology. Chem. Res. Toxicol..

[78] Iqbal Z., Rasool G., Hayat C.S., Akhtar M. (1998). Biochemical Disturbances Associated with Haemonchosis in Sheep. Agric. Sci..

[79] Coop R.L., Kyriazakis I. (2001). Influence of Host Nutrition on the Development and Consequences of Nematode Parasitism in Ruminants. Trends Parasitol..

[80] Mastorci F., Vassalle C., Chatzianagnostou K., Marabotti C., Siddiqui K., Eba A.O., Pingitore A. (2017). Undernutrition and Overnutrition Burden for Diseases in Developing Countries: The Role of Oxidative Stress Biomarkers to Assess Disease Risk and Interventional Strategies. Antioxidants.

[81] Carlsson A.M., Mastromonaco G., Vandervalk E., Kutz S. (2016). Parasites, Stress and Reindeer: Infection with Abomasal Nematodes Is Not Associated with Elevated Glucocorticoid Levels in Hair or Faeces. Conserv. Physiol..

