# Supplementary material for: A Sustainable Strategy for Gastrointestinal Nematode Control in Sheep
Source: Vet Sci. 2026 Jan 21;13(1):104. doi: 10.3390/vetsci13010104 (PMC12846356; doi:10.3390/vetsci13010104)
Supplement: Supplementary file 1 [file vetsci-13-00104-s001.zip › vetsci-4031543-supplementary.pdf]

Day after treatment	Sampling	ID	Number of sampling	Farm	Hair cort.
49	2	1313	1	Bertoni	24,751
49	2	1318	2	Bertoni	17,311
49	2	2285	3	Bertoni	15,324
49	2	3598	4	Bertoni	12,908
49	2	5026	5	Bertoni	39,238
49	2	5037	6	Bertoni	11,445
49	2	5045	7	Bertoni	14,074
49	2	11303	8	Bertoni	6,884
49	2	11312	9	Bertoni	7,182
49	2	19503	10	Bertoni	17,088
49	2	19506	11	Bertoni	27,857
49	2	25773	12	Bertoni	19,036
	2	18305	1	Figaroli	12,443
	2	19830	2	Figaroli	19,246
	2	19832	3	Figaroli	7,737
	2	19835	4	Figaroli	13,205
	2	19837	5	Figaroli	12,626
	2	19838	6	Figaroli	19,112
	2	19840	7	Figaroli	26,724
	2	22419	8	Figaroli	19,448
	2	22420	9	Figaroli	16,921
	2	22430	10	Figaroli	19,382
	2	22433	11	Figaroli	13,313
	2	22434	12	Figaroli	19,893
150	1	1313	3	Bertoni	50,943
150	1	1318	4	Bertoni	39,212
150	1	2285	7	Bertoni	42,961
150	1	3598	9	Bertoni	19,185
150	1	5026	11	Bertoni	37,462
150	1	5037	10	Bertoni	37,462
150	1	5045	12	Bertoni	35,665
150	1	11303	1	Bertoni	18,517
150	1	11312	2	Bertoni	23,918
150	1	19503	5	Bertoni	23,711
150	1	19506	6	Bertoni	48,666
150	1	25773	8	Bertoni	67,539
	1	18305	13	Figaroli	26,032
	1	19830	15	Figaroli	39,388
	1	19832	16	Figaroli	19,141
	1	19835	17	Figaroli	25,43
	1	19837	14	Figaroli	26,845
	1	19838	18	Figaroli	25,235
	1	19840	19	Figaroli	34,458
	1	22419	20	Figaroli	33,356
	1	22420	21	Figaroli	30,193
	1	22433	22	Figaroli	22,65
	1	22430	23	Figaroli	35,568
	1	22434	24	Figaroli	32,582
7	3	1313	1	Bertoni	9,73

7	3	1318	2	Bertoni	20,876
7	3	3598	3	Bertoni	9,342
7	3	5026	4	Bertoni	21,725
7	3	5037	5	Bertoni	8,19
7	3	5045	6	Bertoni	14,421
7	3	11303	7	Bertoni	9,097
7	3	11312	8	Bertoni	9,276
7	3	19503	9	Bertoni	10,22
7	3	19506	10	Bertoni	10,667
	3	18305	1	Figaroli	14,145
	3	19830	2	Figaroli	38,544
	3	19832	3	Figaroli	13,722
	3	19835	4	Figaroli	20,932
	3	19837	5	Figaroli	20,122
	3	19838	6	Figaroli	18,893
	3	19840	7	Figaroli	
	3	22419	8	Figaroli	20,444
	3	22420	9	Figaroli	23,617
	3	22430		Figaroli	17,447
	3	22433	10	Figaroli	13,605
	3	22434	11	Figaroli	19,385

BCS	EPG	level	log EPG	RBC	HCT	HGB	MCV	MCH
3	40	1	1,812913	8,72	26,4	10	30,3	11,5
3	0	1	1,39794	8,13	24,3	8,4	29,9	10,3
3,5	40	1	1,812913	8,83	23,5	9,5	26,6	10,8
3	0	1	1,39794	9,27	30	10,6	32,4	11,4
2,5	200	1	2,352183	8,13	23,6	9,2	29	11,3
3	200	1	2,352183	9,6	29,8	10,4	31	10,8
3,5	920	3	2,975432	9,16	28,4	9,4	31	10,3
3,5	0	1	1,39794	6,74	20,6	9,5	30,6	14,1
3	240	1	2,423246	9,53	24,8	9,1	26	9,5
3	200	1	2,352183	10,35	30,8	10,6	29,8	10,2
2,5	80	1	2,021189	8,86	29,3	9,5	33,1	10,7
3	240	1	2,423246	8,24	30,5	10,1	37	12,3
3	320	2	2,537819	7,86	24,1	9,1	30,7	11,6
3,5	280	1	2,4843	9,83	28,8	9,9	29,3	10,1
3,5	480	2	2,703291	8,16	25	8,8	30,6	10,8
3	400	2	2,628389	9,54	29,3	10,5	30,7	11
3	400	2	2,628389	9,49	27,7	9,4	29,2	9,9
2,5	1240	3	3,102091	7,08	22,4	7,4	31,6	10,5
2	1040	3	3,02735	7,19	24	8,2	33,4	11,4
3	560	2	2,767156	7,96	24,4	8,5	30,7	10,7
3	1680	3	3,231724	6,19	20,2	7,3	32,6	11,8
4	80	1	2,021189	8,57	30,7	9,6	35,8	11,2
2,5	40	1	1,812913	9,48	29,5	10,3	31,1	10,9
2	1560	3	3,200029	8,56	28,8	9,2	33,6	10,7
2,5	240	1	2,423246	8,18	24,5	7,9	30	9,7
2,5	0	1	1,39794	7,02	20,1	6,9	28,6	9,8
2	0	1	1,39794	8,27	21,2	7,5	25,6	9,1
2	1360	3	3,14145	8,06	26,7	8,3	33,1	10,3
2,5	1680	3	3,231724	8,75	25,6	8,5	29,3	9,7
3	40	1	1,812913	7,74	24,5	7,9	31,7	10,2
2,5				7,72	20,8	7,2	26,9	9,3
2,5				8,29	25,5	8,2	30,8	9,9
3				10,24	27	9	26,4	8,8
2	1040	3	3,02735	9,44	28,4	9,2	30,1	9,7
2	40	1	1,812913	8,85	27,2	8,9	30,7	10,1
2	40	1	1,812913	7,55	25	7,9	33,1	10,5
2,5	1240	3	3,102091	9,06	26,4	9	29,1	9,9
2,5	1680	3	3,231724	8,12	27,1	9	33,4	11,1
2,5	1240	3	3,102091	7,11	24	7,7	33,8	10,8
3,5	80	1	2,021189	9,34	30,8	10,2	33	10,9
4	240	1	2,423246	8,82	25,8	8,7	29,3	9,9
3	280	1	2,4843	6,38	20	6,5	31,3	10,2
2	240	1	2,423246	8,92	28,1	9,3	31,5	10,4
3,5	320	2	2,537819	8,58	28,1	9,6	32,8	11,2
2	360	2	2,585461	8	24,8	8,3	31	10,4
3,5	440	2	2,667453	9,85	31,4	10,5	31,9	10,7
3	80	1	2,021189	9,79	35,6	10,9	36,4	11,1
2,5	80	1	2,021189	7,23	24,4	8	33,7	11,1
2	0	1	1,39794	9,8	26,5	9,8	27	10

2	0	1	1,39794	7,8	21,1	7,9	27,1	10,1
2	0	1	1,39794	8,59	26,7	9,1	31,1	10,6
2	0	1	1,39794	8,4	24,1	8,8	28,7	10,5
2,5	0	1	1,39794	9,76	29,2	10,4	29,9	10,7
1,5	40	1	1,812913	8,81	23,5	8,7	26,7	9,9
2,5	0	1	1,39794	9,17	27,4	9,8	29,9	10,7
2	160	1	2,267172	7,2	17,9	0,5	24,9	0,7
2	40	1	1,812913	9,76	27,4	10,5	28,1	10,8
2	0	1	1,39794	10,24	31,4	10,3	30,7	10,1
3,5	280	1	2,4843	10,42	25,1	9,7	24,1	9,3
2,5	1880	3	3,279895	7,31	23	8,1	31,5	11,1
3	200	1	2,352183	7,26	21,3	7,7	29,3	10,6
3,5	40	1	1,812913	10,22	30,8	11,1	30,1	10,9
2	360	2	2,585461	3,47	10,1	4	29,1	11,5
3	80	1	2,021189	6,81	20,9	7	30,7	10,3
3	0	1	1,39794	9,58	27	9,7	28,2	10,1
3,5	240	1	2,423246	9,13	27,3	9,5	29,9	10,4
3	200	1	2,352183	9,32	25,8	9,5	27,7	10,2
4	40	1	1,812913	9,05	32,1	10,4	35,5	11,5
3,5	280	1	2,4843	8,33	27,2	9,3	32,7	11,2
4	40	1	1,812913	9,45	28,5	10	30,2	10,6

MCHC	Reteric	LEU	NEU	LYM	Mono	Eos	Baso	PLT
37,9	1,7	7,96	1,74	4,9	0,57	0,5	0,25	634
34,6	2,4	9,18	1,93	4,41	1,61	1,22	0,01	156
40,4	3,5	9,89	2,44	6,16	0,56	0,46	0,27	734
35,3	4,6	8,71	2,4	4,69	0,89	0,59	0,14	415
39	4,1	7,04	2,25	3,35	1,15	0,24	0,05	409
34,9	0	5,74	1,32	2,55	1,55	0,29	0,03	262
33,1	0,9	5,35	1,24	3,45	0,26	0,4	0	91
46,1	4	11,47	3,74	5,23	0,35	1,78	0,37	1755
36,7	5,7	10,18	2,37	4,75	2,06	0,87	0,13	299
34,4	3,1	5,85	1,19	3,25	0,61	0,58	0,22	155
32,4	2,7	5,2	1,5	2,4	0,57	0,67	0,06	157
33,1	4,1	8,45	2,83	3,86	1,86	0,53	0,17	300
37,8	3,9	8,19	2,96	3,75	0,88	0,5	0,1	813
34,4	10,8	6,14	1,38	3,02	0,72	0,85	0,17	161
35,2	0,8	5,8	1,32	2,56	1,35	0,57	0	190
35,8	2,9	7,42	1,79	4,09	0,77	0,61	0,16	134
33,9	3,8	8,06	2,01	4,22	0,74	1,08	0,01	114
33	7,8	13,33	5,18	6,34	0,59	0,83	0,39	326
34,2	3,6	9,82	4,55	4,02	0,79	0,33	0,13	301
34,8	2,4	6,52	1,33	3,62	0,88	0,59	0,1	150
36,1	1,2	5,71	1,47	3,55	0,47	0,22	0	447
31,3	3,4	6,85	1,86	3,19	1,14	0,58	0,08	110
34,9	3,8	10,18	5,38	3,39	0,61	0,79	0,01	140
31,9	3,4	7,72	2,29	3,59	1,37	0,46	0,01	304
32,2	4,1	8,83	1,88	5,74	0,71	0,33	0,17	140
34,3	4,2	8,96	1,39	5,23	1,63	0,52	0,19	139
35,4	5,8	11,19	2,54	6,08	1,81	0,6	0,16	277
31,1	2,4	9,23	2,8	4,99	0,94	0,35	0,15	166
33,2	4,4	8,51	2,15	4,39	1,66	0,11	0,2	137
32,2	2,3	7,43	1,24	3,65	1,75	0,7	0,9	102
34,6	3,9	7,58	1,51	4,94	0,75	0,3	0,08	162
32,2	5	12,38	3,01	6,62	0,6	1,94	0,21	210
33,3	8,2	11,54	2,27	5,94	2,16	1,01	0,16	158
32,4	4,7	8,29	1,41	4,33	1,52	0,87	0,16	126
32,7	4,4	5,73	1,59	2,65	1,02	0,4	0,07	100
31,6	0	7,64	1,29	4,01	1,93	0,28	0,13	133
34,1	7,2	9,46	3,5	4,19	0,99	0,61	0,17	208
33,2	5,7	6,23	1,29	3,92	0,45	0,48	0,09	132
32,1	3,6	6,05	1,83	2,68	1,17	0,32	0,05	155
33,1	4,7	7,18	1,95	3,75	0,73	0,64	0,11	141
33,7	3,5	8,77	2,6	4,1	1,03	0,92	0,12	196
32,5	4,5	12,13	3,31	6,7	0,75	1,23	0,14	242
33,1	8	7,9	3,06	3,12	1,13	0,56	0,03	180
34,2	0,9	8,24	1,74	4,42	0,9	1,01	0,17	198
33,5	1,6	7,02	1,35	4,37	0,69	0,34	0,27	135
33,4	6,9	10,88	2,79	5,1	1,89	0,98	0,12	108
30,6	6,9	8,74	1,4	4,1	1,3	1,68	0,26	133
32,8	2,2	6,75	1,09	3,22	1,38	0,96	0,1	210
37	2	11,79	2,93	6,01	1,8	0,96	0,09	220

37,4	1,6	14,51	3,48	6,19	2,03	2,79	0,02	287
34,1	0,9	12,56	4,33	5,23	1,47	1,38	0,15	358
36,5	1,7	8,38	2,57	3,25	2,1	0,44	0,02	475
35,6	2	9,94	2,23	5,07	1,35	1,21	0,08	212
37	2,6	7,95	2,03	4,56	0,71	0,65	0	306
35,8	11	17,12	4,77	7,87	1,35	3,12	0,01	341
2,8	2,2	12,21	3,18	5,65	2,43	0,94	0,01	254
38,3	2	11,9	3,16	5,93	0,95	1,59	0,27	510
32,8	3,1	6,61	1,84	2,57	0,76	1,34	0,1	167
38,6	3,1	11,92	2,4	4,94	1,64	2,98	0,03	215
35,2	9,5	6,9	2,47	2,75	1	0,56	0,12	208
36,2	1,5	7,51	1,55	4,01	0,88	1,01	0,06	111
36	4,1	7,63	1,41	4,6	0,31	1,23	0,08	121
39,6	52,1	7,27	3,56	2,22	0,83	0,65	0,01	162
33,5	4,1	15,69	3,16	7,44	1,64	3,31	0,14	320
35,9	1,9	8,85	2,11	4,21	1,38	1,14	0,01	284
34,8	1,8	8,24	1,29	4,76	1,21	0,9	0,08	155
36,8	2,8	8,36	1,32	4,98	0,99	1	0,07	339
32,4	1,8	7,93	1,49	4,05	1,28	1,01	0,1	232
34,2	6,7	10,05	3,32	4,03	1,99	0,71	0	120
35,1	0,9	9,15	2,8	2,96	2,15	1,15	0,09	244

MPV	ALT	AST	BUN	BHBA	Ca	Cl	Cortisol	Creat
11	22	104	8	8,23	10,5	108	1,61	0,9
7,8	21	116	8	7,22	9,2	105	0,86	0,94
10,8	19	107	10	8,46	10,4	104	2,4	0,73
10,4	33	220	5	6,75	9,5	110	1,28	0,72
9,5	29	130	12	7,9	8,7	105	1,78	0,92
9,4								
6,9	26	137	5	7,52	8,7	108	1,23	0,74
13,3	29	107	11	9,25	10,6	106	1,72	0,81
9,3	26	96	15	5,04	9,5	106	0,76	0,82
9,7	22	117	7	7,78	10,4	110	1,44	0,85
8,9	25	115	11	8,75	9,8	108	0,08	0,78
10,4	18	104	10	9,28	9,6	105	1,46	0,68
10,9	32	127	27	5,06	9,5	109	1,72	0,72
9,7	24	73	14	5,22	9,3	111	1,28	0,78
8,5	20	95	14	5,33	9,4	108	1,78	0,59
8,5	25	86	19	3,67	9,9	108	2,57	0,91
8,2	16	80	15	5,9	9,2	110	1,59	0,85
8,6	28	74	17	4,18	9,7	107	3,16	0,83
9,5	24	79	19	15,75	10,1	113	2,13	0,72
8,2	19	84	16	5,21	9,8	108	2,66	0,82
9,8	35	116	13	6,38	9,4	110	2,92	0,76
8,4	17	93	13	4,81	10,2	107	1,14	0,82
8,8	23	92	17	3,54	10,2	107	0,08	0,86
9,4	22	87	15	7,49	10,6	102	0,87	0,88
7,9	16	76	9	5,2	10,7	106	1,82	0,85
9	17	94	13	5,59	9,8	110	3,34	0,97
8,7	19	92	10	6,24	10,7	108	4,53	0,69
7,9	22	110	10	11,85	10,6	106	1,31	0,65
7,7	17	71	7	7,22	9,6	107	0,3	0,84
7,9	21	82	7	8,92	10,7	108	1,19	0,82
8,4	14	72	11	27,9	8,9	106	3,35	0,68
8,1	24	93	12	5,47	10,8	104	2	0,79
8,9	21	96	9	6,93	8,9	110	1,37	0,66
7,8	20	99	7	9,05	10	107	0,79	0,77
9	15	74	9	5,45	10,1	111	1,04	0,87
7	10	71	13	11,65	9,1	109	1,47	0,72
7,6	15	72	20	10,57	9,6	110	3,19	0,88
8,6	20	72	6	5,29	9,3	111	1,75	0,82
8,4	17	84	19	3,27	10	109	2,62	0,81
7,6	21	81	11	5,35	9,8	109	0,84	0,84
7,3	14	76	19	4,32	9,6	106	4,2	0,96
7,7	19	69	28	6,37	9,8	108	2,93	0,7
9,6	15	89	21	15,18	10,5	107	1,92	0,65
7,8	16	75	17	2,97	9,5	105	4,19	0,8
7,6	27	89	27	4,5	9,3	113	3,46	0,72
10,3	20	79	9	7,52	9,3	113	1,61	0,78
8,5	19	91	11	5,09	9,9	108	4,2	0,78
9,2	20	108	25	3,93	10,8	102	3,49	0,72
9,3	25	92	17	5,49	9,8	95	2,53	0,82

8,7	21	119	18	5,96	8,9	95	1,81	0,91
8,9	31	133	27	9,1	9	95	2,89	0,62
8,1	18	92	22	5,9	8,3	95	2,53	0,69
9,1	30	95	18	5,18	9,9	97	2,17	0,83
8,9	24	98	18	5,92	8	98	4,59	0,72
9,5	30	95	24	4,3	9,5	93	2,18	0,9
8,4	24	91	22	5,62	9,3	97	2,27	0,69
8,9	30	116	25	4,47	9,1	97	2,96	0,79
8,9	23	115	27	5,54	9,4	96	1,84	0,73
7,9	28	90	10	3,61	8,9	102	3,98	0,83
11	24	95	5	5,61	8,1	105	0,81	0,69
8,5	24	98	8	3,35	8,6	105	3,11	0,73
8,3	25	94	11	3,83	8,5	101	1,73	0,86
8,5	15	84	18	5,26	7,8	102	2,32	0,73
7,8	24	59	17	3,27	8,8	102	1,45	0,95
8,6	21	85	12	5,92	9,1	102	2,17	0,8
8,2	18	95	12	3,6	8,4	100	1,9	0,94
8,9	39	102	12	5,07	8	103	5,49	0,69
8,6								
10	17	81	7	8,39	9	101	1,97	1,04
8,1	13	63	10	3,8	9,2	100	1,94	0,88

P	NEFA	K	Total Prot	OFR
3,6	342	4,8	7,2	48
4,3	47	4,4	7,5	28
4,3	42	4,3	7,1	37
4,5	177	4,8	6	53
3,4	33	5	7	33
6	47	4,5	7,6	59
4,2	23	4,5	6,9	34
5,9	60	4,5	7,7	32
4	172	4,8	7,6	36
3,5	31	4,5	6,8	33
5,3	28	4,3	6,5	30
3,1	392	4,2	7,3	46
3,6	161	4,4	6,6	47
5,7	52	4,5	6,6	44
3,8	263	5,1	7,5	51
4	3,65	4,6	6,8	55
4,1	65	6	7,6	34
3,5	60	4,6	7,5	60
3	79	4,5	7,7	52
4,1	58	4,7	7,3	35
5,4	269	4,9	6,7	61
4,1	192	4,3	7,5	40
4,4	45	4,6	6,6	62
	33	4,8	8	5
	34	4,8	7,9	4,6
	4	4,5	8,2	51
	50	5,1	7,6	58
	40	5,6	7,5	41
	59	4,9	7,3	42
	71	4,7	7,3	54
	94	5,1	7,7	79
	24	4,8	8,1	74
	32	4,6	7,5	44
	66	4,8	6,6	54
	714	5,6	6,4	76
	881	5,3	5,7	58
	33	5,9	6,9	44
	52	5,2	7	48
	32	5,5	7,7	63
	216	5,8	6,9	47
	591	7,2	7,9	47
	997	5,1	6,9	72
	40	5,4	8	48
	171	4,6	6,6	49
	21	5,4	7,7	69
	51	3,9	6,9	57
	79	5,1	6,8	62
4,8	539	4,5	8,8	40

3,7	39	3,7	8,9	25
3,7	33	4,1	8,6	40
4	34	4,9	7,1	32
4	20	4,5	8,9	40
3,5	23	4,2	8,8	40
4,3	34	4,4	8,4	42
5,4	25	4,6	8,8	21
3,1	35	4,3	8,9	40
4,3	30	3,9	7,8	40
3,1	80	5	7,4	56
2,8	77	4,3	6,4	83
2,8	98	4,5	7,1	21
4,7	136	5	7,7	32
2,7	170	4,9	5	39
3,4	242	6,5	8,2	47
2,7	99	4,4	7,2	17
5,4	395	5,1	8,3	41
2,3	60	4,5	7,6	20
3,2	1162	4,9	6,5	25
5,2	105	5,4	7,4	103
